# Assessment of the magnitude and contributing factors of expired medicines in the public pharmaceutical supply chains of Western Ethiopia

**DOI:** 10.1186/s12913-023-09776-y

**Published:** 2023-07-25

**Authors:** Gamachu Diriba, Gemmechu Hasen, Yesuneh Tefera, Sultan Suleman

**Affiliations:** 1 Pharmaceutical Quality Assurance, and Regulatory Affairs, Madda Walabu University, Goba Referral Hospital, Oromia Regional State, Robe, Ethiopia; 2 Jimma University Laboratory of Drug Quality (JuLaDQ) and School of Pharmacy, Institute of Health, Jimma University, Jimma, Ethiopia; 3 Pharmaceutical Sciences, Pharmaceutical Quality Assurance and Regulatory Affairs, University of Gondar, Gondar, Ethiopia

**Keywords:** Expired medicine, Magnitude, Supply chain, Contributing factors, Western Ethiopia

## Abstract

**Background:**

The magnitude of expired medicines in supply chains are increasing globally due to lack of strict control of the supply chain, poor storage management and oversupply of medicines. This situation is very serious in resource-poor countries, including Ethiopia, where the supply of medicines is limited. Therefore, this study aimed to assess the magnitude and the contributing factors of expired medicines in the Public Pharmaceutical Supply Chains of Western Ethiopia.

**Methods:**

Explanatory sequential study design involving mixed quantitative and qualitative approach were employed among 62 public pharmaceutical supply chains of Western Ethiopia from July1 to August 30, 2021. An observational checklist and the self-administered questionnaire were used to review all records of the expired medicine file and to abstract secondary data on the extent, types of expired medicines and its contributing factors. The collected data was cleared, filtered, and coded using Microsoft Excel® 2010, and exported to SPSS version-23 (Amsterdam, Netherland) for statistical analysis. Bivariate logistic regression was used to check association between the outcome and independent variables. Multivariate logistic regression was analyzed when *p*-value is less than or equal to 0.25 in bivariate binary logistic regression, considering the statistical at *p*-value < 0.05. Moreover, audio recordings were transcribed and coded for emergent themes using thematic analysis.

**Results:**

The study revealed 5% expire rate over past two financial (2012 up to 2013) years and the total amount of expired drugs is estimated at 20 million Ethiopian Birr (ETB). Tetanus antitoxin (TAT), in terms of single drug value, had the highest drug expiry (4,110,426.43ETB: 20%), followed by liquid dosage forms (11,614,266.11 ETB: 57%). The Binary logistic regression result indicated that, poor store management were more likely associated with the magnitude of expired medicine than those with good store management (COR: 10.706, 95% CI: 2.148, 53.348). Multivariate logistic regression revealed that poor store management (AOR: 9.718, 95% CI: 1.474, 64.082) was a significant contributor to the expire rate at 5% (*P* < 0.05). Most facilities did not have a procedure, and programme for disposing of expired medicines. According to key informants, inadequate inventory management, lack of policy and implementation of standards are the main contributing factors of the medicine's expiration.

**Conclusion and recommendations:**

The current study found that the overall rate of medication expiration is high, at a significant cost to the budget. Inadequate inventory management, lack of policy, and implementation of standards are the main contributing factors to the medicine's expiration, as cited by key informants. Further research is necessary to determine the quality and efficacy of these expired drugs to extend their shelf life to ensure adequate access to drugs in resource-limited settings.

**Supplementary Information:**

The online version contains supplementary material available at 10.1186/s12913-023-09776-y.

## Introduction

A medicine should meet the relevant requirements of identity, purity, strength, and quality at the time of use if maintained according to the manufacturer's recommended storage guidelines, which have been defined for each product category [1]. However, in the process of ensuring the availability of medicines to the population, there is a possibility that these medicines may reach such expiration date in health facilities [2]. Globally, the amount of expired medicines is growing at an alarming rate due to lack of effective supply chain management, poor storage management and oversupply of medicines, especially in resource limited setting [3, 4]. Medical advancement has resulted in a significant increase in life expectancy and quality of life. This remarkable development in healthcare has been accompanied by an increase in pharmaceutical waste, owing primarily to an increase in the health seeking behaviour, prescriptions, consumption, and overproduction of medications. As a result, this pharmaceutical waste has created ecological, economic, and ethical burdens that must be understood from various perspectives [5].

The global magnitude of expired drugs increased as a result of a lack of inspection when issuing drugs to stock, a lack of stringent supply chain management, poor storage management, and an excess of drug supply, which increased the burden of expired medicines and their prevalence towards lifesaving drugs [6–8]. As a witness study conducted in China revealed, twenty tons of drugs, 10 tons of medical devices and 724.5 tons of disinfection materials had destroyed because they were inappropriate (mostly expired) and could not be used [9]. Additionally study done in Sri Lanka indicated that the expiry date was not shown on the labels of 50% of the drugs; 6.5% of the medicines expired on arrival, and 67% expired in less than a year [10]. According to a study conducted in India, 72% of people do not check expiration dates when purchasing or taking medications [11].

Expiration of medicines not only affect health factors also it is detrimental for government for loss of money that allocated for health sector. Study conducted in Africa specially in East Africa, money lost due to expired medicines was surprising. As evidence study performed in Rwanda, a study found that the total value of expired medicines was RWF 6,046,778,655, with major contributing factors including supply chain management ranking at 90%, other factors ranking at 73%, poor storage management ranking at 68%, and excessive drug supply ranking at 67% [12]. Also, the study conducted in South Africa explored the total estimated value of financial losses due to medicine expiry was R 836 029; an estimated annual revenue loss of 0.6% of the total hospital expenditure that contributed to lack of standard treatment guideline knowledge among prescribers [13]. A part, study finding from Uganda, and Tanzania depicted that, medicines expired due to delivering minimum shelf life not specified in order (23.7%), using neither FIFO nor FEFO in stock management (5.3%), and overstocking of medicines (32.4%) [14], and receiving near expiry date (61.1%) & over stocking of the items as the main cause of product expiration [15].

Globally, the availability of expired medicine in stores of supply chain and health facility is enormous [5, 12, 13, 16]. Literatures explored medicine expired at different level of health facility and in different kinds. However, there are few studies examining general issues of expired medicine in public pharmaceutical supply chains but same related studies investigated medicines wastage (due to expiration) in community settings either as home storage or returns to pharmacies [17, 18]. From expired medicine point of view, most studies conducted in Ethiopia were concentrated at household level rather facility level. Due to poor governance, poor documentation, a lack of auditing procedures, transparency, and accountability in the pharmaceutical system of the country were possible to found expired drug in the health facilities which increases healthcare costs and depletes the nation’s insufficient health system resources [3, 19].

None of the research showed investigated the distribution of pharmacological categories of discarded medications at the supply chain level, however the study conducted in Ethiopia emphasised the extent of pharmaceutical waste in healthcare institutions. As proof, a study carried out at six institutions in the town of Gondar found that the assessed value of expired single-drug essential medicines was expressed in terms of money (26,760 ETB), rather than pharmacological categories [20]. This study demonstrates the extent of expired medications and the factors that contribute to them at the pharmacological category level, which is necessary for forecasting studies. Additionally, the study conducted in public health facilities in East Shewa zone, Oromia regional State, revealed that a total of 174,366.98 Ethiopian birr was lost in health facilities because of expired medicines. Also, associated factors such as receiving near expiry products (55%), oversupply of medicines (45%), insufficiency of store rooms (75%), and failure to apply FEFO principles in some facilities (25%), were found to be the common problems leading to medicine waste [21]. According to a study done by Gurmu T. G. and Ibrahim A. J. in 2017, the total monetary value of wasted medicines in the surveyed health facilities in EFY 2005–2007 was 500,522.09 Ethiopian Birr, while in the same period, the wastage rate was 7.5% [17]. A descriptive qualitative study conducted by Alnahas F et al. in 2020 reported that of a total of 70 programme commodities managed by the agency, 2.1% were wasted due to expiration and damage. These resulted in a loss of over $2 million [18].

According to the study, there was a significant amount of medicine waste in Ethiopia that requires immediate attention and action. Because medicine expiration is a universal problem in the pharmaceutical supply chain, strong policymakers and programme-supporting agencies must design an appropriate system for reducing the extent of expiration, which leads to the loss of useful medicine, economic expenditure, environmental concerns, antibiotic resistance, and improving overall supply system productivity [16, 22, 23]. More than half of the people in some of Africa's poorest countries, such as Ethiopia, do not have regular access to essential medicine [24]. In addition to making medicines inaccessible, the continued accumulation of expired medicines creates administrative burdens and can endanger the environment and public health [5].

This study was the first of its kind in pharmaceutical supply chain history to examine the extent, and the contributing factor of an expired drugs in the Western Cluster at supply chain level. One reason for it was the fact that no prior research had been done in Ethiopia's Western Cluster, where large populations and subpar supply chain management are located [25, 26]. The district Regional Health Bureau regularly discards medications from public health institutions in the western portion of Ethiopia; nevertheless, the type and extent of medication expiry as well as its underlying causes were not looked into. A different study conducted in Ethiopia demonstrated that no study has yet revealed objective evidence on the type and magnitude of expired medicines in the Western cluster of the pharmaceutical supply chain in the region [8, 22, 27]. Taking the aforementioned issue into consideration, the study aims to assess the magnitude of expired medicines and contributing factors in western Ethiopia's public pharmaceutical supply chain.

### Methods and materials

#### Study area, and period

The study was conducted in the western cluster of the Ethiopian Pharmaceutical Supply Agency (EPSA) and its service delivery points and health facilities from July 1 to August 30, 2021. EPSA is a governmental organisation mandated to provide affordable and quality pharmaceuticals sustainably to public health facilities. EPSA is a government organisation tasked with providing affordable and high-quality pharmaceuticals to public health facilities on a long-term basis. EPSA's headquarters are in Addis Ababa, where the organisation's nineteen branches and seven clusters are coordinated. The Western Cluster, comprised of three branches such as Jimma, Nekemte, and Gambella, served as a hub for governmental structures for the implementation of an integrated pharmaceutical logistics system and the distribution of essential health commodities for public and private health facilities located in seventeen zones, two special woredas, and one town administration, serving approximately 8,591,795 people living in the catchment area. The organisation also provides supervision, material support, and capacity building to health facilities in order to strengthen and enforce IPLS and pharmacy service implementation [28]. As no studies have been done in the region, the study may focus on the types, extents, and contributing causes of expired pharmaceuticals in Western Ethiopia in order to inform policymakers and programme agencies and establish appropriate practises to reduce the extent of drug expiration. This is due to the substantial population and intricate supply structure in Ethiopia's western area [26, 29]. A recent agency programme called branch clustering sought to provide effective services by encouraging mutual cooperation among branches based on proximity, which opens the door for easier access to performance reports and other information via the three clusters. The branches of Jimma, Nekemte, and Gambella make up the western EPSA cluster. These branches are in charge of providing medications to linked healthcare establishments [26].

### Study design

An explanatory sequential study design was employed in the public pharmaceutical supply chain of western Ethiopia. First, the quantitative data was collected and analysed. Then, qualitative data was collected and analysed based on the quantitative findings. For qualitative study, in depth face-to-face interview was conducted by principal investigator with general director, pharmacy head, and store manager to fully explore in-depth information about the current situation of expired medicines, factors contributing to the expiration of medicines, and possible intervention mechanisms to reduce the extent of expired medicines.

### Source and study population

All of the public pharmaceutical supply chains found in western cluster of Ethiopia were used as the source of population.

### Study population

Public pharmaceutical supply chains of western cluster of EPSA branch, Hospitals, Health centre and selected health management Information system like Health Commodity Management Information System (HCMIS), DAGU, records of expired medicines, disposal reports, EPSA Distribution file, model 19 and all inventory management records of the past two fiscal year (2012-2013EC) in the sampled health facilities were the study population. All pharmacy head, general director and store manager in the sampled health facilities was the study population.

### Sampling techniques

In the western cluster of pharmaceutical supply chain agencies, a total of 369 healthcare facilities were found that involved hospitals (*n* = 30) and health centres (*n* = 339). It was challenging to address these healthcare facilities. Therefore, for general representation of the study site, a simple random sampling technique was utilised using the Logistics Indicators Assessment Tool (LIAT) [20]. The study was carried out at two EPSAs (*n* = 2), nine hospitals (*n* = 9), and 51 health care facilities (*n* = 51) selected by simple random sampling technique. For the qualitative study, the general director, pharmacy head, and store manager in the selected facilities were purposefully selected as key informants because they are supposed to be more information-rich than other health professionals. The number of key-informants was determined depending on the saturation of information concerning emerging themes.

### Sample size

The sample size of public pharmaceutical supply chains in western Ethiopia was calculated using the Logistics Indicators Assessment Tool (LIAT) [20]. The document recommends that a minimum of 15% of the targeted health facilities be included in the survey. Accordingly, from three western cluster hubs of the Ethiopian pharmaceutical supply agency, two (70%) of them were selected. Then, from the total number of hospitals (30) and health centres (339) available in the study area, nine (30%) hospitals and fifty-one (15%) health centres were selected. The total sample size of the study was 62 (See supplementary material).

## Inclusion and exclusion criteria

### Inclusion criteria

All public pharmaceutical supply chains established in the western cluster before to three years, all pharmacy professionals who present at the study site and volunteer to participate in the study during the study period, and all expired medicines recorded with prices in the previous two fiscal years (2012–2013) were included.

### Exclusion criteria

Expired medicines that are recorded as free, such as programme and donation medicines were excluded. In addition, nongovernmental pharmaceutical supply chains such as the Red Cross, private and wholesale pharmacies, as well as drug stores, were excluded.

### Study variables

#### Independent variables

Facility demographic variables, personnel demographic variables, documents and records, management of pharmaceutical inventory (scheduled and unscheduled procurement), management systems, level of store management (good and poor), updated and outdated essential drug lists, standard treatment guidelines (updated and outdated), and good and poor accountability of store managers were grouped as independent variables.

#### Dependent variables

The magnitude of expired medicines, and the level of medicine expiration (high and low) could be included as independent variables.

### Data collection tools

An observational checklist was used for the quantitative study to review all records of the expired medicine file and to abstract secondary data on the extent and types of expired medicines. The expired medicines checklist was generated using the EFMHACA medicines waste management, and disposal directive data collection sheet [29]. The self-administered questionnaire was adapted from Nakyanzi et al [30] for collecting of information from socio demographic profiles, and the contributing factors of expired medicines. The handling protocol of expired medicines was prepared based on the EFDA medicines waste management, and disposal directive.

The key informants are selected purposefully based on their richness of information regarding factors contributing to the expiration of medicines, the extent of expired medicines, and the possible intervention mechanisms being followed for the reduction of expired medicines in the health facilities because they are familiar with pharmaceutical supply chain information. The qualitative data collected through face-to-face interviews with key informants using a semi-structured interview guide that was developed from the literature review [13, 15, 17].

Two pharmacists and the principal investigators were allocated as data collectors for quantitative and qualitative data respectively. The quantitative data were collected using a standard checklist through observation, document review from HCMIS, and the expired medicines register book. The qualitative data collection technique was used a semi-structured interview guideline. Key informant interviews, were conducted with the chief pharmacist and general directors about the overall picture of the magnitude of expired medicine by the principal investigator (See Supplementary materials). Interviews were prepared in English, then translated to Afaan Oromo and Amharic. The principal investigator was spent an average of 30 min in-depth interviewing the key-informants. The principal investigator also used the reflexivity method to improve the rigour of the data collection, which enabled better probing, fewer assumptions, the avoidance of premature interpretation, and an accentuated sense of curiosity during the interview.

### Data quality assurance

The study questionnaire was developed after reviewing different literatures, and Pharmaceutical Supply chain agency guideline, published in English language. Strategies followed for assurance of the quality of mixed data were triangulation, prolonged engagement with data, persistent observation, negative case analysis, and referential adequacy are all procedures that can be used to increase the credibility of quantitative, and qualitative studies. Then, data was coded, checked for accuracy, consistency, omissions, and irregularities, and then prepared for analysis from all selected supply chains using validated data collection forms. Prior to data collection, a data collector is trained for one day on the data collection instruments and processes.

To ensure the reliability of the study of the self-administered questionnaires and respondent’s understanding of the questions, questionnaire was pretested before actual data collection with 16% of total sample size of the study, which not included in the study area. The reliability of the self-administered questionnaire was assessed with the Cronbach's alpha coefficient of 0.723. The principal investigator was assigned to supervise the data collection process, and any inconsistencies were corrected as soon as possible.

The interview guide for an in-depth interview was tested for face-to-face and content validity by experts from the pharmaceutical quality assurance and regulatory affairs group. It was written in English, translated into Amharic, and then returned to English to ensure message consistency (See Supplementary materials). Additionally, mobile audio recorder was used to record the interview and filed notes on important points. Appropriate personal protective equipment (PPE), such as sanitizer and a face mask, was used by the data collectors and the study participants before undergoing data collection. Pharmaceutical quality assurance, regulatory affairs, and pharmaceutical supply chain professionals reviewed the validity and reliability of self-administered questionnaires. The self-administered questionnaires include pharmacy heads and pharmacy store managers because these are specialists whose work is somewhat closely tied to medication management.

### Data management and analysis

For the quantitative study the collected data was classified, filtered, and coded using Microsoft Excel® version 2010. The information was then exported to statistical package for Social Sciences (SPSS) version-23 (Amsterdam, Netherland) for statistical analysis. The Microsoft excel was used for analysis of the quantitative result of the magnitude of expired medicines, and the estimated money value, unit pack, dosage form, types and extents of expiration rate in each health facility. The handling protocol and the contributing factors of expired medicines were analysed using the statistical package for social sciences. Binary logistic regression was used to determine the association between dependent and independent variables while multivariate logistic regression was used to explore major contributing factors. Statistically significant difference was considered when *p* < 0.05.

The storage management associated with the expiration of medicine was also classified as poor store management, and good store management, which contributed to medicine expiration in health facilities. These criteria were adopted from Ethiopian Pharmaceutical Supply Agency monitoring and evaluation manual and logistics indicator assessment tool (LIAT) [31, 32]. The facility fulfilment of the storage condition, those facilities that fulfilled the criteria more than/equal to 80% were considered as good, whereas, those fulfilled less than 80% were considered poor [31]. Based on the country's current drug availability and drug information, the model list of essential drugs was also divided into updated and outdated categories. An auditing of the national essential medicines list for medicines that have been withdrawn or authorised for the market and those that are currently on the model were categorised as outdated, and updated lists, respectively [33]. The principal investigator guaranteed the acceptance requirements, and country medicine authorities released recommendations.

For the qualitative study, the principal investigator performed face-to-face in-depth interviews to explore the factors responsible for the expiration of medicines and possible intervention mechanisms to reduce the extent of the drug expiration rate in the public pharmaceutical supply chains. The investigator transcribed the audio recordings of in-depth interviews and discussions verbatim. Audio-recordings and notes were translated into English. Transcribed data were coded and analysed for emergent themes using thematic analysis, as per the approach and steps recommended by Braun and Clarke [34]. The number of key informants is due to the saturation of information concerning emerging themes. Then, codes were given, issues were discussed, and themes were developed. Later, the text relating to each code/theme was discussed and summarised in subthemes, and the findings were presented using quotes.

Qualitative data were analysed using deductive content analysis techniques, thematic contents were formulated, and a master list of themes was developed based on the research questions and conceptual framework. Then, the data analysis was employed by developing codes from the texts, and they were categorised as the following terms: current situation of expired medicines, factors that contributed to expired medicines, and intervention mechanisms employed to reduce the extent of expired medicines. The themes that developed from the codes would be supplemental for reasons for expired medicines and factors contributing to expired medications. Each transcript was carefully screened and triangulated with the quantitative result.

## Results

### The background information of the health facilities profile

From the total of 62 public pharmaceutical supply chains included in the study, sixty (*n* = 60) facilities were successfully assessed, with a 96% response rate. From these, 45 (75%) of the facilities received a score greater than two which indicating high expiration rates, while 15 (25% of them) received a score less than two which indicating low expiration rates. Among the public pharmaceutical supply surveyed to assess the magnitude of expired medicine and contributing factors, 2 (3.3%) EPSA, 8 (13.3%) hospitals, and 50 (83.3%) health centres in western Ethiopia had expired medicines. From the total of 60 facilities surveyed, 47 (78.3%) were established before 10 years, and 52 (86.7%) had no excess budget for drug procurement. Of the total public pharmaceutical facilities assessed, there were three (*n* = 3) health professionals with different educational levels under the pharmaceutical store manager. The general information of the public pharmaceutical facility profile was depicted in Table 1.

**Table 1:** Frequency of background information of the health facilities

S. No	Variables	Categories	Frequency (*n* = 60)	Percentage (%)
1	Facility	Health centre	50	83.3%
		Hospitals	8	13.3%
		EPSA	2	3.3%
2	Year of established	Less than 5 years	-	%
		5- 10 years	13	21.7%
		More than 10 years	47	78.3%
3	Has the facility extra budget for drug procurement	Yes	8	13.3%
		No	52	86.7%
4	Professions of Store Manager	Druggist/Pharmacist	41	68.3%
		Nurse	13	21.7%
		Midwife	6	10%
		HO	-	%
5	Age	20–30	35	58.3%
		30–40	24	40%
		Above 40	1	1.7%
6	Gender	Male	35	58.3%
		Female	25	41.7%
7	N of experiences	1–5	39	65%
		5–10	20	33.3%
		Above 10	1	1.7%
8	Highest Educational Level	Diploma	36	60%
		Degree	23	38.3%
		MSc and above	1	1.7%
9	Expire Rate depends on EFDA Standard	Fail	45	75%
		Pass	17	25%

### Magnitude of expired medicine

**Fig. 1:**
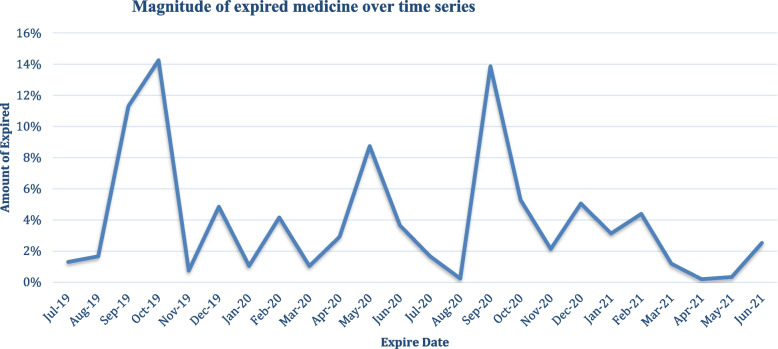
Magnitude of expired medicine by date of expires

In the public pharmaceutical supply chain of western Ethiopia, a total of two hundred thirty-nine thousand and eight hundred one (*n* = 239,801) unit packs that consist of different anatomical therapeutic classifications (ATC) and dosage forms that are estimated to cost more than twenty million birr (= 20,538,198.93 ETB) of money value expired over the study period from August 2012 to June 2013 E.C. Regarding the availability of expired medicines, 45 (75%) of the supply chains had more than two expired medicines, and 15 (25%) had less than two expired medicines. The details of the magnitude of expired medicines in the respective health facilities, expired cost in Birr, purchased cost in Birr, and expired rate were presented in the Table 2. As can be seen in Fig. 1, the magnitude of expired medicine was high (14%, 13%) in January 2019 and November 2020, relative to the percentages in July 2019 and May 2021, respectively.

**Table 2:** Magnitude of expired medicines at western Ethiopia

S.No	**Health Facility Code**	**Unit Pack**	**Expired cost in Birr**	**Purchased cost in Birr**	**Expire Rate (%)**
1	HC-1	437	34,693.14	274,573.86	12.63
2	HC-2	317	11,464.51	123,580.9506	9.27
3	HC-3	181	15,416.12	166,588.17	9.25
4	HC-4	303	22,069.41	254,649.33	8.66
5	HC-5	337	13,273.73	184,245.95	7.2
6	HC-6	347	13,897.01	198,569.64	6.9
7	HC-7	381	8404.32	120,661.55	6.9
8	HC-8	319	8784.72	150,294.99	5.8
9	HC-9	229	11,158.09	198,723.88	5.6
10	HC-10	267	7364.64	133,677.29	5.5
11	HC-11	273	7403.6	135,309.95	5.47
12	HC-12	184	11,155.6	220,775.4	5
13	HC-13	159	12,288.03	259,930.74	4.7
14	HC-14	175	12,241.38	260,699.5	4.6
15	HC-15	128	7925.07	180,300.52	4.3
16	HC-16	389	8237.7	189,518.59	4.34
17	HC-17	287	8532.55	201,404.4	4.2
18	HC-18	302	15,506.07	378,384.49	4.09
19	HC-19	711	18,102.89	446,037.45	4.05
20	HC-20	82	3776.21	100,518.22	3.7
21	HC-21	177	5605.63	171,233.84	3.2
22	HC-22	70	6021	196,763.6	3
23	HC-23	213	8571.79	309,206.81	2.7
24	HC-24	348	10,153.19	367,292.22	2.6
25	HC-25	241	13,508.11	523,860.03	2.5
26	HC-26	179	5440.37	218,395.84	2.4
27	HC-27	63	4159.24	171,921.97	2.4
28	HC-28	87	4993.38	210,382.93	2.37
29	HC-29	191	6184.19	261,654.12	2.36
30	HC-30	204	15,652.05	673,326.55	2.3
31	HC-31	75	2895.54	128,556.24	2.25
32	HC-32	803	8365.23	377,563.32	2.2
33	HC-33	266	11,944.37	554,890.23	2.1
34	HC-34	203	10,012.02	472,551.6	2.1
35	HC-35	129	3078.03	148,367.81	2
36	HC-36	135	5200.47	254,461.4	2
37	HC-37	53	2470.84	121,085.1	2
38	HC-38	77	4910.8	243,742.91	2
39	HC-39	108	3371.72	175,433.47	1.92
40	HC-40	145	6737.36	358,895.2	1.87
41	HC-41	154	6586.99	411,993	1.5
42	HC-42	83	5187.03	352,157.81	1.4
43	HC-43	281	8512.34	581,515.17	1.46
44	HC-44	98	3294.78	227,918.57	1.44
45	HC-45	173	3877.13	287,346.19	1.34
46	HC-46	89	2907.64	220,996.66	1.31
47	HC-47	183	4770.57	367,029.33	1.29
48	HC-48	84	4508.49	358,470.2	1.25
49	HC-49	84	3246.4	294,109.32	1.1
50	HC-50	43	2944.76	452,300.92	0.65
51	H-1	1526	60,806.32	671,904.88	9
52	H-2	4069	186,600.89	3,886,644.73	4.8
53	H-3	1779	24,184.25	862,179.78	2.8
54	H-4	997	36,990.18	4,276,796.98	0.86
55	H-5	960	32,684.32	4,203,634.34	0.77
56	H-6	410	22,549.93	3,532,062.36	0.63
57	H-7	400	36,039.27	5,810,205.42	0.62
58	H-8	3737	96,939.62	23,210,651.12	0.41
59	EPSA-1	154,572	15,305,058.86	322,746,862.62	4.7
60	EPSA-2	60,504	4,293,310.47	51,762,098.16	8.29
**Total**		**239,801**	**20,538,198.93**	**434,634,907.6**	**5%**

The expiration rates in the public pharmaceutical supply chain of western Ethiopia were 5%, 1%, and 3% in the Ethiopian pharmaceutical supply agency (*n* = 2), hospitals (*n* = 8), and health centres (*n* = 50), respectively. In relative terms, the magnitude of expired medicine appears to be best in hospitals, poor in health centres, and least in EPSA (Table 3).

**Table 3:** Level of supply chain with magnitude of expired medicine

S/No	Indicators for magnitude of expired medicine	Level of public pharmaceutical supply chain		
		**EPSA (*N* = 2)**	**Hospital (*N* = 8)**	**Health centre (*N* = 50)**
1	Money value (ETB)	19,598,369.33 (95%)	496,794.78 (3%)	436,806.25 (2%)
2	Quantity of unit pack	215,076.0 (90%)	13,878 (6%)	10,847 (4%)
3	Expiration rates in each supply chain (%)	5%	1%	3%
4	Highest dosage form (%)	57% Liquid	56% Liquid	62% Solid
5	Highest class of drugs (%)	21% Vaccine	13% Cardiovascular Drugs	29% Anti-Infective
6	Highest single drugs (%)	21% Tetanus Antitoxin 1,500 IU/1 ml Ampoule	5% Tetanus Antitoxin 1,500 IU/1 ml Ampoule	3% Glucose 40% Injection

**Fig. 2:**
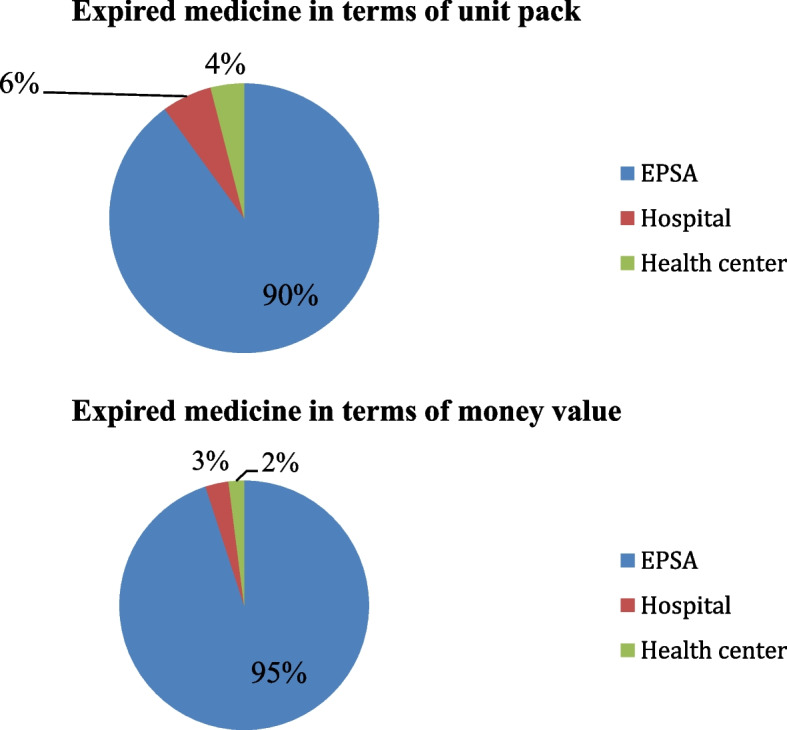
Magnitude of expired medicine by level of supply chain

When compared by level of supply chain, the magnitude of expired medicines from total expired cost was 90%, 6%, and 4% in terms of unit pack, and 95%, 3%, and 2% in terms of money value in EPSA (*n* = 2), hospitals (*n* = 8), and health centres (*n* = 50), respectively (See in Fig. 2).

### Types of expired medicine by anatomical therapeutic class

As can be seen in Table 4, drugs used in vaccines (28%; 5,729,098.86 Birr) and drugs used in anaesthesia (19%; 3,822,248.71 Birr) constituted the highest classes of expired medicines, followed by those used as anti-infectives (18%; 3,652,829.93 Birr) and central nervous system drugs (17%; 3,470,925.94 Birr). Only 8% (1,667,104.28 ETB) of expired electrolyte medicines were recorded. Based on the ATC classes, ophthalmic agents (3% (615,538.27 ETB), cardiovascular drugs (3% (581,226.14 ETB), gastrointestinal system 2 (485,900.47 ETB), dermatologic agents (1% (207,105.67 ETB), and blood products (0.5ectives (18%; 3,652,829.93 Birr) and central nervous system drugs (17%; 3,470,925.94 Birr). Only 8% (1,667,104.28 ETB) of expired electrolyte medicines were recorded. Based on the ATC classes, ophthalmic agents (3% (615,538.27 ETB), cardiovascular drugs (3% (581,226.14 ETB), gastrointestinal system 2 (485,900.47 ETB), dermatologic agents (1% (207,105.67 ETB), and blood products (0.5%) (79,473.57 ETB) were found to be expired, respectively.

**Table 4:** Expired medicine by anatomical therapeutic class

S.No	Anatomical Therapeutic Class	Amount In Birr	Percent
1	Vaccine	5,729,098.86	28%
2	Drugs Used in Anaesthesia	3,822,248.71	19%
3	Anti-Infective	3,652,829.93	18%
4	Central Nervous System Drugs	3,470,925.94	17%
5	Electrolyte	1,667,104.28	8%
6	Ophthalmic Agents	615,538.27	3%
7	Cardiovascular Drugs	581,226.14	3%
8	Gastrointestinal System	485,900.47	2%
9	Dermatologic Agents	207,105.67	1%
10	Blood Products	79,473.57	0.5%

### Types of expired medicine by unit packs of ATC

From the total unit packs of medicines that were expired (239,801.0), about 94,909 (40%) were found to be used in both local and general anaesthesia. Central nervous system drugs 27,951 (12%), anti-infective 26,166 (11%), electrolyte 21,313 (9%), ophthalmic agents 14,501 (6%), gastrointestinal system 13,663 (6%), cardiovascular drugs 11,491 (5%), ear, nose, and throat preparations 8567 (4%), drugs used in endocrine disorders 7907 (3%), and dermatologic agents 6068 (3%) expired during the study period (See in Table 5).

**Table 5:** Anatomical therapeutic class of expired medicine by quantity of unit packs

S. No	ATC	Quantity of Unit Pack	Percent
1	Drugs Used in Anaesthesia	94,909	40%
2	Central Nervous System Drugs	27,951	12%
3	Anti-Infective	26,166	11%
4	Electrolyte	21,313	9%
5	Ophthalmic Agents	14,501	6%
6	Gastrointestinal System	13,663	6%
7	Cardiovascular Drugs	11,491	5%
8	Ear, Nose, And Throat Preparations	8567	4%
9	Drugs Used in Endocrine disorders	7907	3%
10	Dermatologic Agents	6068	3%

### Types of expired medicine by dosage forms

**Fig. 3:**
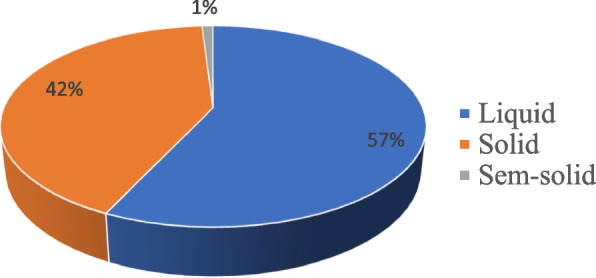
Expired Dosage forms in terms of money value

**Fig. 4:**
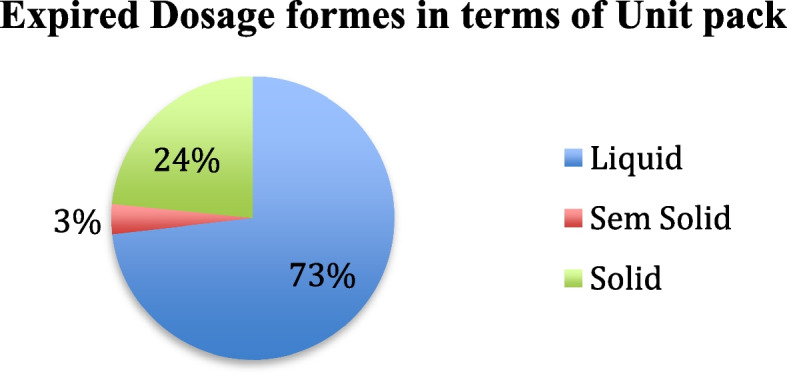
Expired Dosage forms in terms of Unit pack

Based on the dosage form, 175,513 (73%) unit packs that cost 11,614,266.11 ETB (57%) were expired drugs, of which 56,137 (24%) unit packs were liquid and 8,723,541.62 ETB (42%) were solid, and the remaining 8154-unit packs (3%) were equivalent to 194,336.27 ETB (1%) semisolid dosage forms found to be expired (See in Figs. 3, and 4).

### Magnitude of expired medicine with highest cost single drugs

**Fig. 5:**
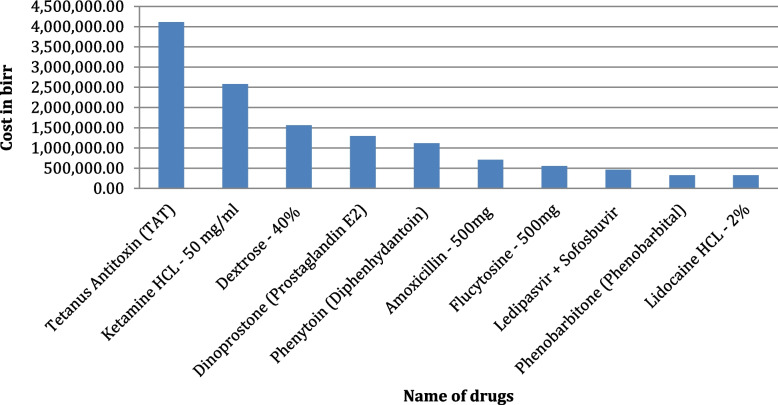
Top ten (*n* = 10) highest cost of expired medicine

As can be shown in Fig. 4, medicines were also ranked by their expired cost. Accordingly, tetanus antitoxin (TAT), Equine—1,500 IU/ml in 1 ml Ampoule that accounted to 4,110,426.43 (20%) birr were the highest drug that was expired followed by Ketamine HCl—50 mg/ml in 10 ml that costs 2,581,497.60 ETB (13%) and Dextrose—40% in 20 ml—Intravenous Infusion 1,561,966.36 (8%) was expired over the study period (See in Fig. 5).

### Handling protocol of expired medicine

The majority of facilities (60%) have the necessary records and documentation for expired drugs and a separated and secured storage system. However, the majority of the facilities did not have procedures or programmes for disposing of expired medicine. Therefore, expired drugs were reimbursed or were at risk because of the high expiration rate and the lack of proper disposal mechanisms. Expired drugs were stored for a long time without disposal, and returned medicines were disposed of poorly as per the EFDA procedure and recommendation at the facility (See in Table 6).

**Table 6:** Handling protocol of expired medicine

S/N	**Handling for expired drug**	**Degree of Agreement**				
		SA	A	N	D	SD
1	Facilities have necessary records and documentation for expired drugs	8% (*N* = 5)	60% (*N* = 36)	25% (*N* = 15)	7% (*N* = 4)	-
2	Storage of Expired drug are separated from unexpired and secured	25% (*N* = 15)	27% (*N* = 16)	18% (*N* = 11)	13% (*N* = 14)	7% (*N* = 4)
3	Have Procedure and programs for disposal of expired medicine	2% (*N* = 1)	2% (*N* = 1)	15% (*N* = 9)	55% (*N* = 33)	27% (*N* = 16)
4	Expired Drugs are reimbursed or at risk because of high expired rate and luck of proper disposal	17% (*N* = 10)	37% (*N* = 22)	22% (*N* = 13)	20% (*N* = 12)	5% (*N* = 3)
5	Expired Drugs stored for long time without disposal and returned back at the facility	18% (*N* = 11)	28% (*N* = 17)	28% (*N* = 17)	20% (*N* = 12)	5% (*N* = 3
6	Expired drug disposed as EFDA recommendations at the facility	8% (*N* = 5)	15% (*N* = 9)	27% (*N* = 16)	22% (*N* = 13)	28% (*N* = 17)

NB: *SD* Strongly agree, *A* Agree, *N* Neutral, *D* Agree, *SD* Strongly disagree

### Major contributing factors for magnitude of expired medicine

Binary logistic regression was employed to determine the association between independent variables and outcome variables. The Binary logistic regression result indicated that, poor store management were more likely associated with the magnitude of expired medicine than those with good store management (COR: 10.706, 95% CI: 2.148, 53.348). In the current study, binary logistic regression indicated a strong association between the magnitude of expired medicines, and independent variables indicative of the most contributing factors for the magnitude of expired medicines over the past two financial years (2012 up to 2013 EC) in the health facility. However, the multivariate logistic regression explores that, poor store management was the major contributing factors to the magnitude of expired medicine (AOR: 9.718, 95% CI: 1.474, 64.082) at a 5% (*P* ≤ 0*.*05) in the health facilities. The summary of the results of the logistic regression of associated factors responsible for the magnitude of expired medicines is indicated in Table 7.

**Table 7:** Major contributing factors for magnitude of expired medicine

S.No	Variable	Category	Magnitude of Expired medicine		COR (95% CI)	AOR (95% CI)	*P*-value
			High	Low			
1	Schedule for Procurement	Scheduled	6 (10%)	6 (10%)			
		Non-scheduled	39 (65%)	9 (15%)	4.333(1.13–16.612)	2.715(.430- 17.136)	0.67
2	Store Management	Good	17(28.3%)	13(21.6%)			
		Poor	28(46.6%)	2(0.1%)	10.706(2.148–53.348)	9.718(1.474- 64.082)^*^	0.03*
3	Essential drug list	Updated	6 (10%)	6 (10%)			
		Outdated	39 (65%)	9 (15%)	4.333(1.13–16.612)	.311(.051–1.891)	0.91
4	STG	Updated	4(6.67%)	4(6.67%)			
		Outdated	41(68.4%)	11(18.3%)	3.72(.801–17.4)		0.61
5	Accountability of store manager	Good	10(16.6%)	8(13.3%)			
		Poor	35(58.3%)	7(11.6%)	4(1.164–13.74)	1.383(.308- 6.224)	0.92

* significant difference, CI Confidence Interval, *COR* Crude Odd Ratio, *AOR* Adjusted Odd Ratio

## Qualitative Results

### Background information of the key informant

Key informants were asked about the factors responsible for the expiration of medicines and possible intervention mechanisms to reduce the extent of drug expiration rates in the public pharmaceutical supply chains. The issues explored included the current situation of expired medicines in the public pharmaceutical supply chain, factors that contributed to expired medicines, and intervention mechanisms employed to reduce the extent of expired drugs. The majority of interviewees (*n* = 12; 80%) were male, with (*n* = 11; 73%) having their first degree, (*n* = 13; 87%) having 1–5 years of experience in their position, and (*n* = 11; 73%) serving as head in their position. Fifteen (100%) of the participants were qualified pharmacists. All individual participants were drawn from the total sample size of the study (60) in the western public pharmaceutical supply chain, as shown in Table 8.

**Table 8:** Background information of the key informant

S. No	Back ground Information	Category	Frequency (*N* = 15)	Percentage (100%)
1	Age	20–30	4	27%
		30–40	8	53%
		Above 40	3	20%
2	Sex	Male	12	80%
		Female	3	20%
3	Highest level of education	Diploma	2	13%
		Degree	11	73%
		MSc Degree	3	20%
4	Total Work experience at position	1–5	13	87%
		Above 5	2	13%
5	Current position in the health facility	Pharmacy Unit	3	20%
		Pharmacy Head	11	73%
		General Director	1	7%

From the total interview conducted, the following information was gathered about expired medicine, which was categorised into three main themes: the current situation of expired medicines in the public pharmaceutical supply chain, factors that contribute to the expiration rate of medicines, and possible intervention mechanisms to reduce the extent of expired drugs.

### Current situation of expired medicines

The majority of the interviewers answered the question, "How do you assess the current situation of expired medicines in your facility?" Medicine expiration is a common occurrence, but program drugs are more likely to have expired than those obtained from the public supply chain. However, they had expired medicine that they procured from EPSA that expired because of low turnover and a short expiration date while being delivered.

*“Program drugs are at risk because they have expired; due to the facility has no stores for expired program drugs or disposal schedules, and also, distributors are not collecting back the expired medicine*." *"Moreover, procured drugs expired in almost small amounts, which resulted in the relatively small expenditure of our budget for the procurement of drugs (CP1)."*

One of the things negatively affected by drug expiry as a result of the loss of useful medicine is expenditure." *Furthermore, there is no evidence of a drug-free environment, which is associated with drug resistance. We were simply burning and planting expired drugs in fields to dispose of them. "The expired drug disposal mechanisms currently used may be risky for future generations (CP4)."*

*“The extent of expired medicines is reduced by working collaborating with store manager. However, it is difficult to prevent medicine expiration having short expiration dates, and EPSA program have near expiration dates (CP8).”*

More than half of Respondents Answer the questions, “how an expired medicine affecting service provision of the given facility? deliberately loss of therapeutically useful drugs because of expire affects the economics of health facility as well as lack of secured and enough store that resulted to environmental hazards and makes additional workload on the disposal of expired medicines.

### Factors that contributed to expired medicines

For the question, "What are the factors that contribute to expired medicines in your facility?" All respondents listed the factors responsible for the expiry of medicines based on their experiences. Almost all respondents agreed on poor turnover, a short expiration date (which is almost going to expire), higher delivery from higher supply, poor forecasting by EPSA Head Office during procurement, and poor quantification of facilities during selection. Furthermore, as mentioned by key informants, poor inventory management systems at health facilities and a lack of policy and standard implementation were factors that contributed to medicine expiry at their respective facilities.

*“Poor turnover is the most important factor responsible for expired medicine in health facilities. Poor turnover may be caused by prescribing medications without following standard treatment guidelines and having an out-of-date essential drug list with current disease prevalence (CP3’’).*

*Receiving short shelf-life products from EPSA with no other option was the main reason for the presence of high expire rate of medicine in the facility's high expiry rate." "EPSA never takes stock out to buy long shelf-life medicines from private sources, so the received near-expired medicine, resulting in a high expiry rate or expenditure due to expired medicine (CP11’’).*

As interviewers mentioned from EPSA, factors contributing to expired medicines are mainly sourced from Central EPSA due to poor inventory management, poor forecasting during procurement from manufacturers, which resulted in the pushing of short shelf-life products to branches and may cause expired medicines in the public pharmaceutical supply chain.

*“At the level of Central EPSA procurement system poor; forecasting, quantification, and inventory Management systems were among the possible factors responsible for high degree of expiry rate of medicines in branch warehouses (CP10).”*

*There was a high magnitude of expired medicine was found in the warehouse because of customer need fluctuation and poor quantification at the service delivery point, and due to receiving of the short shelf-life products from central EPSA that increase the expiry rate of medicines in the western branch (CP15)."*

The majority of participants forwarded almost the same idea for the question, "Are there any efforts made so far by the facility to prevent or reduce expired medicines?" No efforts were made by the facility, but as a pharmacy unit, they tried all the necessary efforts to improve inventory management by using the FIFO mechanism all the time and redistributing their overstocked and slow-moving items to other facilities based on their need for the product. At EPSA, there is an inventory improvement team to prevent and minimise the extent of expired medicine.

*“Lack enforcement of FIFO system and procuring near expire medicines, which are commonly associated to high expiry rate (CP2)’’.*

*“Having team that works on improvement of inventory management process such as schedule for physical count, updating stock cards, arranging products on shelf and supporting store manager to minimize the expiry rate of medicines at the facility (CP6).”*

Key-informants remind us, in order to reduce the amount of expired medication, the pharmacy unit is making all necessary steps to improve inventory management. Despite the fact that pharmacy professionals work tirelessly to address the issue of the loss of useful medications due to expiration, nearly half of them were answered as a lack of effort by facility management and a sizable burden or obligation placed solely on the store manager.

### Possible intervention mechanisms to reduce extent of expired medicine

All respondents mentioned their recommendations for reducing the extent of expired medicine, and nearly all responded similarly to the question, "What do you recommend to minimise the magnitude of expired medicine in the future?".

*“Properly use of FIFO (first in first out) mechanism is vital action in prevention of expenditure due to expire (CP8).”*

*“Avoiding unqualified professional workers at position of pharmaceutical store manager (CP 7).’’.*

*Developing policy and regulation regarding the extent of medicine expire to increase accountability of facility administrators and employers who work on medicine management (CP9).”*

*“Developing policy and regulation to facilitate redistribution of near expire and slow-moving medicine and also electronic based reports for information exchange between heath facility that facilitate the need to accept and donate (CP12).’’.*

*"Strong coordination between all warehouses and perfect inventory management systems at the facility's pharmaceutical stores may prevent the expenditure of useful medication due to expiration (CP14)."*

## Discussion

In the study, the magnitude of expired medicines, and the contributing factors to expiration were assessed in the western part of Ethiopia based on the country's pharmaceutical supply chain cluster, which considered unit packs, money value, and expiration rate, as well as the contributing factors that fast-track the expired medicines in the respective study area. From the public pharmaceutical supply chain of western Ethiopia (EPSA, hospitals, and health centres), a total of two hundred thirty-nine thousand and eight hundred one (*n* = 239,801.0) expired unit packs were revealed. The finding showed that, too many unit packs consisted of strips of tablets, capsules, vials, ampoules, tubes, tins, and bottles of syrup, which lead to environmental hazards if not managed and disposed of appropriately. This report was inconsistency with study conducted in south Africa which found 32,368 unit packs [13] and at Awi zone, Amhara regional state, a total 605 medications were found expired at the pharmacy stores of those hospitals [22]. The difference may be related to the sample size of the study, in which the current study assessed 60 public pharmaceutical supply chains in western Ethiopia, including EPSA, hospitals, and health centres, while comparative studies assessed single facilities or a few.

Many pharmaceuticals, without a doubt, save lives and alleviate suffering, but some high-volume drug distribution may harm the environment as well as the government's health budget(38). As a result, despite the rules, a lot can and does go wrong, including inappropriate or expired medicines discovered in public health facilities [22]. The expiration rates in the public pharmaceutical supply chain of Western Ethiopia were 5%, 1%, and 3% in Ethiopian pharmaceutical supply agencies (*n* = 2), hospitals (*n* = 2), and health centres (*n* = 50), respectively. In relative terms, the magnitude of expired medicine appears to be best in hospitals, poor in health centres, and least in EPSA. Also, the study found that a total of 15 different anatomical therapeutic classes (ATC) of expired medicine, including, anti-infective (18%) and central nervous system medicines (17%), were the most common expired in public facilities. The presence of more expired anti-infective drugs may create a favorable environment for the possibility of bacterial resistance if improperly disposed of, and drugs for the central nervous system may lead to reuse and misuse because they contain narcotic drugs [31]. Similar findings were reported from a study conducted in South Africa, which revealed that of approximately 68 different anatomical therapeutic chemical (ATC) classes, antibacterial for systemic use (16%) were the most common expired medicines, followed by antiviral medicines used for systemic use (15%) [13].

The causes and economic consequences of expired medicine, despite the fact that medicine costs account for a significant portion of healthcare expenditure. Medicines and their administration are critical health-care functions that are required for health improvement and maintenance [13, 32]. However, the lack of essential medicines and the regulatory system that governs them remains one of the most serious public health issues, as well as a major source of health budget allocation worldwide, including in Ethiopia [33]. The study found that an expired medicine had an estimated value of $20 million (20,538,198.93 ETB) in western Ethiopia. It demonstrates a significant financial outlay that expired medicine is complicated, and it may result in an administrative burden. This result is higher than that of a study conducted in the West Wollega Zone, where the total value of reproductive health medicines thrown away due to expiration was 357,920.52 ETB (12,323.81 US dollars) [34], and Shoa Zone, Oromia regional state, the total monetary value of wasted medicines was 500,522.09 ETB [17]. However, it was lower than a study conducted in Ruanda, which revealed that the total value of expired products for all program categories was RWF 6,046,778,655 [12]. Pharmaceuticals that have passed their expiration dates endanger the environment as well as the healthcare system. Given the financial restrictions on funding the healthcare system, as well as the enormous quantity of medicine waste and disposal expenses in low- and middle-income nations, these issues pose a serious danger to both the healthcare system and the overall economy.

In the current study, an aggregated 5% expiration rate was found, which indicates the presence of a high magnitude of expired medicines. Identical reports were posted from some studies done in Africa [12, 13, 17]. However, the current study's aggregated expire rate was lower than that of Ethiopia's national assessment report from 2003, which revealed a total of 8% expire rate in the same year. The variation made due to level of pharmaceutical supply chains and sample size. Also, the majority 45 (75%) of public pharmaceutical supply chains of westerns had expiry rates greater than two, which mean unacceptable range, with comparing to Ethiopian Minister of health, that recommend the maximum of two medicine wastage rate. The efficacy of drugs should be taken into account in this context from a clinical and pharmacological perspective. This illustrates the drug's capacity to have the desired effect [35]. Therefore, better results are obtained with more efficacy. Due to decreased efficacy, medication that has expired may not successfully cure minor ailments (such as a slight headache or cold) or major conditions (such as diabetes or heart disease). Therefore, insufficient recovery from illness could eventually result in more absences from work or school, lengthier sick leaves, and decreased productivity. These challenges have caused us to be concerned about the frequency of outdated medication in healthcare facilities and to look for solutions.

In order to make it simple to estimate the amount of expired drugs in the public pharmaceutical supply chain, the active ingredients in the Anatomical Therapeutic Chemical (ATC) classification system are divided into various groups in the study based on the organ or system on which they act as well as their therapeutic, pharmacological, and chemical properties [36]. The study depicted that, vaccine was the most common expired medicines (28%) followed by anaesthesia (19%, and ant infective (18%) respectively. These three classes of expired medicines may lead to bacterial resistance when they are disposed of poorly, and expired anaesthesia may be used in criminal action if stored in an open field without attention. Furthermore, the study discovered that vaccines (28%, and liquid 57% with high degradation) had the most expired drug classes and dosage forms. Because liquid medications are typically mixed with preservatives, when the expiration date is reached, the preservatives cease to function properly and the chemical compositions of the drugs begin to degrade, posing serious health risks [37].Vaccines could also be subject to quick degradation once the expiration date is reached [38].

Regarding anti-infective, similar study conducted in Tanzania which showed that, anti-infective medicines (18%) were the most common expired medicines next to antibacterial drugs [15]. According to Trueman et al.study's, gastrointestinal medicine (12.4%) was the most commonly detected expired medicine, followed by skin (11.2%), pain medication (10.5%), and cardiovascular medicines (10.3%). According to Glanville J et al.reports from the United States in 2010, pain relievers were the most commonly un sued medications (15%), followed by antibiotics (6.7%), cardiovascular medications (9.7%), medication for gastrointestinal problems, and skin infection both accounts (5.2%) [39]. The difference may be due to economic conditions, as the developed countries have stronger supply chain system than developing country like Ethiopia.

When there is a concern about expired medication storage, patients and family members need clear instructions on how to dispose of it [23]. The actual study evaluated the expired medicine handling protocol; the majority of them lacked disposal procedures or programs and were stored for an extended period of time before being returned. As a result, expired medicine is either reimbursed or put at risk due to a lack of proper and timely disposal. This contradicts the Ethiopian Food, Medicine, and Healthcare Administration and Control Authority's recommendation that medicines that are no longer fit for use be stored for no more than six months [29]. When expired pharmaceuticals are improperly stored and disposed of, they can lead to contamination and a wide range of toxicities in man and animals [40]. People can be exposed or accumulate traces or residues of pharmaceuticals from the environment by drinking contaminated water. Additionally, expenditure of useful medicine due to expire can cause both economical implication and lack of essential medicine. Therefore, it is recommended in the FDA guidelines for drug disposal that expired medicines should not be stored for a long time in a facility store [41]. According to several research, storing unneeded or leftover medications for an extended period of time may cause polypharmacy or inadvertent medication ingestion, which can have hazardous effects on people [42]. In order to prevent medication stock-over, for healthcare providers, training and clinical notation should be provided on a regular basis.

Identifying the factors that contribute to the path of expired medicines in the public supply chain is an important step toward maintaining and reducing the number of expired medicines [3]. The result of the multivariate logistic regression of the study showed that unscheduled procurement, poor inventory management, an out-of-date essential drug list, and poor accountability of the store manager to reduce the extent of expired medicine at the facility were significantly associated with the magnitude of expired medicine. The finding of the study was identical with the reports from South Africa were poor quantification and forecasting and poor inventory management [13] also study conducted in Uganda supply outlets revealed, irrational procurement, provision of medicines not based on the needs, and requisition as well as negligence of stock monitoring [30]. Also the study some extent identical with study employed in Rwanda which revealed that poor storage management, and excessive drug supply were the major the contributing factors for the expiration of medicines [12]. However, the study was differ from study done in Tanzania were over stocking, and pilferage are the most common contributing factors for the expiration of medicines [15]. The difference may be due to the variation of the supply chain system, and inventory management system the facilities were used. Literature has described the immediate effects of inappropriate drug supply and demand, but there are also risks associated with improper disposal and storage practises. Accidental intoxication, incorrect self-medication, pharmaceutical compounds in waterways as environmental contaminants, accidental wildlife poisoning, and the threat of antibiotic resistance are a few examples that come to mind in this context [43–45].

The qualitative aspects of the study also revealed factors that contribute to expired medicines as: Poor turn over, short shelf life (which is almost going to near expire) delivery from higher supply and poor quantification of facilities during selection. additionally, weak pharmaceutical supply chain system and lack of implementations of policy and standards was contributed for the expiration of medicine as cited by key-informants. This was in concordance with study conducted at South west shoa zone of Oromia regional state evidenced that poor quantification practices and donors ordering large quantities of drugs without collaborating with the departmental user inputs contributed to large quantities of expired drugs [17]. Moreover, the study revealed that specific contributed factors to EPSA was due to pushing of short shelf-life product to branches at central EPSA were mainly cause of expire in public pharmaceutical supply chain of western. This was constant with previous literature said that supply chain management related factors (selection, ordering, and supply planning), excess drug supply and short shelf life of medicines affect the expiry of medicine [12]. Therefore, a solid supply chain management system should be installed at the healthcare facility to track store-related variables [46].

The study noted that, the current situation of medicine expire was not good that resulted from misuse, inaccessibility of essential medicine and revenue loss due to expiration. In addition, a lack of secure and adequate storage facilities created environmental hazards and increased the workload on staff to dispose of them [47]. Given the economic loss, this was a significant challenge. Because the majority of medicines that expire in hospitals are essential medicines, reducing waste may help to improve access and availability [13] and the finding was consistent with other studies that found essential medicines had the highest rate of expiry [30]. The findings also revealed a possible intervention mechanism to reduce the extent of expired medicine by key informants' recommendations, which were proper use of the FIFO mechanism, avoid distribution of short shelf-life drugs, strong coordination between warehouses, and perfect inventory management at pharmaceutical stores [48].

Furthermore, it was critical to develop policy and regulations regarding medicine expiration in order to increase the accountability of facility administrators, store managers, and employers who value medicine management [13]. The study was conducted in conjunction with Sauls C's (2013) report, which stated that "sound coordination and communication between the pharmacy and other departments, therapeutics committees should emphasize the use of STG, rigorous vigilance in medicine management, and maintenance of demand planning at all levels of the supply chain may improve stock management. Similarly to what the study suggests, a multidisciplinary team should convene during quantification to collaborate products with a short shelf life should be procured in quantities to be directly distributed to clients to be used before their expiration, and well-managed drug supply chain management may reduce the frequency and quantity of expired drugs [12]. Many low and middle income countries should strengthen public systems for medicines management in order to improve inventory control and the reliability of procurement forecasts, as well as reduce stress on central medical stores through liberalization and reimbursement schemes. Also, to reduce the extent of expired medicines, the national government is responsible for developing expiry date extension methods and designing policies for proper disposal [49].

### Strength and limitation of the study

The study's strengths were its unwavering response to the observed gap and its thorough response to the research questions. Due to financial restrictions, the study only focused on public pharmaceutical supply chains in western Ethiopia, leaving out health posts and private pharmaceutical service providers. As a result, it is challenging to generalise the findings to all pharmaceutical supply chains in the entire region. Since the dataset included medications in a variety of formulations, it was more difficult to estimate the number of expired medications; as a result, the proportion of expired medications was calculated using the price of the expired medication.

## Conclusion and recommendation

The study made an effort to determine the magnitude and contributing factors to the expiration of medicine in the western Ethiopian pharmaceutical supply chain. An aggregate of 5% of expired medicines were found in the pharmaceutical supply chain of western Ethiopia, which evidenced the presence of a high magnitude of expired medicine. The study showed that poor store management, policy, and standards were the major contributing factors to medicine expiration at the health facility level as cited by key informants. A significant number of the facilities lacked procedures or programmes for getting rid of leftover medications. Due to the high rate of drug expiration and the absence of appropriate disposal methods, expired medications were either reimbursed or put in danger. According to the study, in order to ensure effective delivery of pharmaceutical items, supply chain agencies should pay attention to the money lost when medicine warranties expire.

Because expired medicines and their contributing factors are a dynamic phenomenon in pharmaceutical supply chain systems, they were not completely controlled. Hence, all pharmaceutical supply chain agencies at the federal and regional levels have to pay attention and implement robust inventory management system for effective supply of medicines at health facilities.

## Supplementary Information

**Additional file 1. **Data Collection Tools.

**Additional file 2: S1 File. **Sample size calculation.

**Additional file 3: S2 File. **Data Quality Assurance.

## Data Availability

The supporting documents for this study were available within the manuscript and attached as supplementary files.

## Abbreviations

ATC: Anatomical Therapeutic Classification
EPSA: Ethiopian pharmaceutical supply agency
FIFO: First in First Out
HCMIS: Health Commodity Management Information System
LIAT: Logistics Indicators Assessment Tool

## References

[1] Farrugia CA. Controlled temperature storage of medicinals: good practice measures in the community pharmacy. University of Malta. J Malta Coll Pharm Pract. 2005.

[2] World Health Organization (2008). Essential medicines Biennial Report. The pharmaceutical scene in he pharmaceutical scene in 2006–2007.

[3] Gebremariam ET, Gebregeorgise DT, Fenta TG (2019). Factors contributing to medicines wastage in public health facilities of South West Shoa Zone, Oromia Regional State, Ethiopia: a qualitative study. J Pharm Policy Pract.

[4] Chris M, Kong F, Ngeh S, Yagnik P, Peersman G, Ostrum B, et al. Year one evaluation report - medical supply reform impact evaluation Papua New Guinea. 2013;(December):1–80.

[5] Alnahas F, Yeboah P, Fliedel L, Abdin AY, Alhareth K. Expired Medication : Societal , Regulatory and Ethical Aspects of a Wasted Opportunity. Int J Environ Res Public Heal. 2020. 10.3390/ijerph17030787 PMC7037917 32012703

[6] Kebede O, Tilahun G, Feyissa D (2021). Storage management and wastage of reproductive health medicines and associated challenges in west Wollega zone of Ethiopia: a mixed cross-sectional descriptive study. BMC Health Serv Res.

[7] Shukar S, Zahoor F, Hayat K, Saeed A, Gillani AH, Omer S (2021). Drug Shortage: Causes, Impact, and Mitigation Strategies. Front Pharmacol.

[8] Alemu AB, Ibrahim NA. Magnitude of Medicine Wastage and Perceived Contributing Factors Among Public Health Facilities in Dire-Dawa City Administration , in Mid COVID-19 Pandemic in Ethiopia: Retrospective , Cross-Sectional Study. 2023;(March). 10.2147/IPRP.S395102 PMC10028296 36960433

[9] van Dijk DP, Dinant GJ, Jacobs JA. Inappropriate drug donations: What has happened since the 1999 WHO guidelines? Educ Heal Chang Learn Pract. 2011;24(2). 22081650

[10] Mahmood M, Riley K, Bennett D, Anderson W. The Supply of Pharmaceuticals in Humanitarian Assistance Missions : Implications for Military Operations. 2011;176(August):852–7. 10.7205/milmed-d-10-00259 21882772

[11] Kozak MA, Melton JR, Gernant SA, Snyder ME (2016). A needs assessment of unused and expired medication disposal practices: A study from the Medication Safety Research Network of Indiana. Res Soc Adm Pharm.

[12] Hakuzimana T, Kayumba PC, Hahirwa I, Kabalisa M (2021). Assessment of Factors Contributing to Medicine Expiry in Rwanda: Case of the Medical Procurement and Production Division. Rwanda J Med Heal Sci.

[13] Sauls C (2013). Trend in revenue loss due to expired medication at a large urban hospital in Johannesburg, South Africa.

[14] Nakyanzi JK, Kitutu E, Fadhiru P. Lessons from the field Expiry of medicines in supply outlets in Uganda. Bull World Health Organ. 2010;(February). 10.2471/BLT.08.057471 PMC2814474 20428373

[15] Kagashe GA, Makenya FB, Buma D. Medicines Wastage at a Tertiary Hospital in Dar Es Salaam Tanzania. 2014;4(06):98–102.

[16] Tull K (2018). Drug expiry standards in developing countries Question • What is the global evidence/experience on expiry of drugs?.

[17] Gebremariamdis ET (2017). Assessment of Medicines Wastage and Its Contributing Factors in Selected Public Health Facilities in South West Shoa Zone, Oromia Regional State, Ethiopia.

[18] Boche B, Mulugeta T, Gudeta T (2020). Assessment of Inventory Management Practices at the Ethiopian Pharmaceuticals Supply Agency, Addis Ababa Ethiopia. Integr Pharm Res Pract.

[19] YewbneshAlemayehu B (2017). Assessment on Auditable Pharmaceutical Transactions and Services Implementation Outcome: the Case of Amanuel Mental Specialized Hospital a Thesis Submitted To School of Graduate Studies of St. Mary University in Partial Fulfillment of the Requirement for.

[20] Fentie M, Somasundaram J (2015). Availability of Essential Medicines and Inventory Management Practice in Primary Public Health Facilities of Gondar Town. North West Ethiopia J PharmaSciTech.

[21] Gurmu TG, Ibrahim AJ (2017). Inventory management performance of key essential medicines in health Inventory management performance of key essential medicines in health facilities of East Shewa Zone, Oromia Regional State. Ethiopia Cukurova Med J.

[22] Ebrahim AJ, Teni FS, Yimenu DK. Unused and Expired Medications: Are They a Threat? A Facility-Based Cross-Sectional Study. J Prim Care Community Heal. 2019;10. 10.1177/2150132719847857 PMC6537296 31072253

[23] Bashaar M, Thawani V, Hassali MA, Saleem F (2017). Disposal practices of unused and expired pharmaceuticals among general public in Kabul. BMC Public Health.

[24] Mathewos Oridanigo E, Beyene Salgedo W, Gebissa Kebene F. Affordability of Essential Medicines and Associated Factors in Public Health Facilities of Jimma Zone, Southwest Ethiopia. Adv Pharmacol Pharm Sci. 2021;2021. 10.1155/2021/6640133 PMC7987439 33817643

[25] Boche B, Temam S, Kebede O (2022). Inventory management performance for laboratory commodities and their challenges in public health facilities of Gambella Regional State, Ethiopia: A mixed cross-sectional study. Heliyon..

[26] Gizaw T, Bogale M, Gudeta T (2021). Investigating the effect of pharmaceutical logistics service performance on customer satisfaction: a two-step approach with structural equation modeling. J Pharm Policy Pract..

[27] Tura AJ, Dalecha DD. Expiry of medicine in public health facilities of Arsi Zone , Oromia Regional State , Ethiopia: a quantitative and qualitative study. 2022;(August).

[28] EPSA. Western Cluster - Ethiopian Pharmaceuticals Supply Agency - EPSA.

[29] Haji M sani K (2020). The effect of supply chain integration on operational performance of Ethiopian Pharmaceutical supply Agency Jimma Branch.

[30] Nakyanzi JK, Kitutu FE, Oria H, Kamba PF. Expiry of medicines in supply outlets in Uganda. Vol. 88, Bulletin of the World Health Organization. 2010. p. 154–8. 10.2471/BLT.08.057471 PMC2814474 20428373

[31] EFMHACA. Medicines Waste Management and Disposal Directive. Addis Ababa; 2011

[32] Systems for Improved Access to Pharmaceuticals and Services (SIAPS) Program. Promising practices: warehousing and inventory management; 2014:1–16. 2014;2014

[33] List of Essential Drugs for Ethiopia: six Edition. 2020.

[34] Virginia Braun & Victoria Clarke. Using thematic analysis in psychology, Qualitative Research in Psychology. 2006;3(2):77–101. 10.1191/1478088706qp063oa.

[35] Serwecinska L (2020). Antimicrobials and Antibiotic-Resistant Bacteria. Water.

[36] Serwecinska L. Antimicrobials and Antibiotic-Resistant Bacteria. Water. 2020;12:1–17.

[37] Gumas ED. U.S. Health Care from a Global Perspective, 2022: Accelerating Spending, Worsening Outcomes. 2023.

[38] Suleman S, Woliyi A, Woldemichael K, Tushune K, Duchateau L, Degroote A, et al. Pharmaceutical Regulatory Framework in Ethiopia: A Critical Evaluation of Its Legal Basis and Implementation. Ethiop J Health Sci. 2016;26(3):259–76. 10.4314/ejhs.v26i3.9 PMC4913194 27358547

[39] Kebede O, Tilahun G, Feyissa D. Storage Management and Wastage of Reproductive Health Medicines in Public Health Facilities of West Wollega Zone, Ethiopia: Mixed Study. Res Sq. 2019. 10.1186/s12913-021-06291-w PMC8017678 33794887

[40] Wen H, Jung H, Li X (2015). Drug Delivery Approaches in Addressing Clinical Pharmacology-Related Issues: Opportunities and Challenges. AAPS J.

[41] Rashighi M, Harris JE (2017). The Need for New Approaches in CNS Drug Discovery: Why Drugs Have Failed, and What Can Be Done to Improve Outcomes. Physiol Behav.

[42] Serafini M, Cargnin S, Massarotti A, Tron GC, Pirali T, Genazzani AA (2021). What’s in a Name? Drug Nomenclature and Medicinal Chemistry Trends using INN Publications. J Med Chem.

[43] Van Dijk D, Dinant G-J, Jacobs JA, Dpj D, Inappropriate JJ, Drug Donations: What has Happened Since the,  (1999). WHO Guidelines?. Education for Health.

[44] Publishing HH (2019). Drug Expiration Dates — Do They Mean Anything_ - Harvard Health.

[45] Glanville J. Evaluation of the Scale , Causes and Costs of Waste Medicines Evaluation of the Scale, Causes and Costs of Waste Medicines.

[46] Michael I, Ogbonna B, Sunday N, Anetoh M, Matthew O (2019). Assessment of disposal practices of expired and unused medications among community pharmacies in Anambra State southeast Nigeria: A mixed study design. J Pharm Policy Pract.

[47] Societies RC, Federation IP (1999). Guidelines for Safe Disposal of Unwanted Pharmaceuticals in and after Emergencies.

[48] Bashatah A, Wajid S. Knowledge and disposal practice of leftover and expired medicine: A cross-sectional study from nursing and pharmacy students’ perspectives. Int J Environ Res Public Health. 2020;17(6). 10.3390/ijerph17062068 PMC7142560 32244973

[49] Law AV, Sakharkar P, Zargarzadeh A, Tai BWB, Hess K, Hata M (2015). Taking stock of medication wastage: Unused medications in US households. Res Soc Adm Pharm..

[50] Huang H, Li Y, Huang B, Pi X (2015). An optimization model for expired drug recycling logistics networks and government subsidy policy design based on tri-level programming. Int J Environ Res Public Health.

