# Supplementary material for: Assessment of the magnitude and contributing factors of expired medicines in the public pharmaceutical supply chains of Western Ethiopia
Source: BMC Health Serv Res. 2023 Jul 25;23:791. doi: 10.1186/s12913-023-09776-y (PMC10367394; doi:10.1186/s12913-023-09776-y)
Supplement: Supplementary file 1 — Additional file 1. Data Collection Tools. [file 12913_2023_9776_MOESM1_ESM.docx]

**Data Collection Tools**

**Part-I: Facility and personnel demographic Profiles**

1. Health Facility level: Health center Hospital EPSA
2. Year of established: Less than 5 years Between 5 to 10 years

More than 10 years

1. Has the facility additional budget for drug procurement? Yes No
2. Procure near expire drug obligatory from EPSA? Yes No
3. Professions of Store Manager: Drug/ Pharm Nurse

Midwifery HO

1. Age : _______________________in years
2. Gender : Male Female
3. Number of years of experiences in current job position: __________in years
4. Highest Educational Level: Diploma Degree Msc PhD

**Part-II: Checklist for evaluation of supply chains regarding handling of expired medicine.**

1 (Strongly Agree), 2 (Agree), 3 (Neutral), 4 (Disagree), 5 (Strongly Disagree)

| S/N | **Protocols for expired drug** | Degree of agreement | | | | |
| --- | --- | --- | --- | --- | --- | --- |
|  |  | 1 | 2 | 3 | 4 | 5 |
| 1 | Necessary records for expired drugs |  |  |  |  |  |
| 2 | Storage of Expired drug are separated from unexpired |  |  |  |  |  |
| 3 | Procedure and programs for disposal of expired medicine |  |  |  |  |  |
| 4 | Expired Drugs are reimbursed or at risk |  |  |  |  |  |
| 5 | Expired Drugs stored for long time without disposal |  |  |  |  |  |
| 6 | Dispose as EFDA recommendations |  |  |  |  |  |

**Part-III: Checklist for evaluation of supply chains regarding magnitude of expired medicines.**

| **S.N** | **Drug name and strength** | **ATC** | **Quantity** | **Unit** | **Ex Date** | **Value** | **P.V** |
| --- | --- | --- | --- | --- | --- | --- | --- |
|  |  |  |  |  |  |  |  |
|  |  |  |  |  |  |  |  |
|  |  |  |  |  |  |  |  |
|  |  |  |  |  |  |  |  |
|  |  |  |  |  |  |  |  |
|  |  |  |  |  |  |  |  |
|  |  |  |  |  |  |  |  |
|  |  |  |  |  |  |  |  |
|  |  |  |  |  |  |  |  |
|  |  |  |  |  |  |  |  |
|  |  |  |  |  |  |  |  |
|  |  |  |  |  |  |  |  |
|  |  |  |  |  |  |  |  |
|  |  |  |  |  |  |  |  |
|  |  |  |  |  |  |  |  |
|  |  |  |  |  |  |  |  |
|  |  |  |  |  |  |  |  |
|  |  |  |  |  |  |  |  |
|  |  |  |  |  |  |  |  |
|  |  |  |  |  |  |  |  |
|  |  |  |  |  |  |  |  |
|  |  |  |  |  |  |  |  |
|  |  |  |  |  |  |  |  |
|  |  |  |  |  |  |  |  |
|  |  |  |  |  |  |  |  |
|  |  |  |  |  |  |  |  |
|  |  |  |  |  |  |  |  |
|  |  |  |  |  |  |  |  |
|  |  |  |  |  |  |  |  |
|  |  |  |  |  |  |  |  |

| **S.N** | **Variables** | 1 | 2 | | 3 | 4 | 5 |
| --- | --- | --- | --- | --- | --- | --- | --- |
|  | **Pharmaceutical Inventory management** |  |  | |  |  |  |
| 1 | Schedule for Procurement |  |  | |  |  |  |
| 2 | Selection and quantification during procurement is depend on EDL |  |  | |  |  |  |
| 3 | Near expiry drugs procuring |  |  | |  |  |  |
| 4 | Utilisation of STG in the facility |  |  | |  |  |  |
|  | **Administrative system** |  |  |  | |  |  |
| 5 | Financing system for drug Procurement |  |  | |  |  |  |
| 6 | Coordination with other supply chain stuff |  |  | |  |  |  |
| 7 | Facility service delivery |  |  | |  |  |  |
| 8 | Have Pharmacy professional on store manager position |  |  | |  |  |  |
|  | **Store** |  |  |  | |  |  |
| 9 | Storage management |  |  | |  |  |  |
| 10 | Monthly physical count |  |  | |  |  |  |
| 11 | Use FEFO mechanisms |  |  | |  |  |  |
| 12 | Status of Bin and stock cared |  |  | |  |  |  |
|  | **Implementation of policy and guideline** |  |  | |  |  |  |
| 13 | Have awareness of drug policy |  |  | |  |  |  |
| 14 | Status of essential drug list at the facility |  |  | |  |  |  |
| 15 | Status of STG at the facility |  |  | |  |  |  |
| 16 | Use Information system and Necessary software at the facilities |  |  | |  |  |  |
|  | **Other** |  |  | |  |  |  |
| 17 | Regulation enforcement to medicine management |  |  | |  |  |  |
| 18 | Effort/accountability of store manager to reduce expired medicine |  |  | |  |  |  |

**Pat-IV: Guide for evaluation of supply chains regarding factors contributed to exp. Medicine**

1. (Strongly Agree), 2 (Agree), 3 (Neutral), 4 (Disagree), 5 (Strongly Disagree)

**Part-V:Guide for key informants interview/discussion points for qualitative study**

**1. Back ground information of the key informant**

1. Age______________
2. Sex______________
3. Highest level of education ______________
4. Total Work experience ______________
5. Current position in the health facility ______________

**2. Guiding questions for in-depth interview with chief pharmacist at facility**

1. How do you assess the current situation of expired medicines in your facility?
2. What are the factors that contribute for expired medicines in your facility?
3. How an expired medicine affecting service provision of your facility?
4. Are there any efforts made so far by the facility to prevent/ reduced expired medicines?
5. What do you recommend to minimize magnitude of expired medicines for the future?

**Stratified sampling frame**

**8 Hospital**

**47 Health center and**

**1 EPSA**

**56 Supply chain**

**Total health facility**

**26 Hospitals,**

**312 Health Centres**

**Seven Cluster of EPSA**

**Three Hubs of Western Cluster**

**Total health facility**

**4 Hospitals,**

**27 Health Centres**

**Jimma**

**Gambella**

**Total sample size=62**

**9 Hospital**

**51 Health center and**

**2 EPSA**

**6 Supply chain**

**1 Hospital**

**4 Health center and**

**1 EPSA**

**Catchment Profile of Western Cluster of EPSA and service delivery point.**

| S.N | Branch | Distance from  A. A | Region | Available Zones | Available woredas | Hospitals | Health Center | 30 % of Hsp | 15% of HC |
| --- | --- | --- | --- | --- | --- | --- | --- | --- | --- |
| 1 | Jimma | 358 KM | Oromia | 8 | 81 | 26 | 312 | 8 | 47 |
| 2 | Gambella | 714 KM | Gambella | 3 | 14 | 4 | 27 | 1 | 4 |
| Total sample size of Hospital (30 %) | | | | | | | | 9 |  |
| Total sample size of health center (15 %) | | | | | | | |  | 51 |
| EPSA Branch (70 %) | | | | | | | | 2 |  |
| Total sample size of the study will be | | | | | | | | | 62 |
