# Supplementary material for: Assessment of the magnitude and contributing factors of expired medicines in the public pharmaceutical supply chains of Western Ethiopia
Source: BMC Health Serv Res. 2023 Jul 25;23:791. doi: 10.1186/s12913-023-09776-y (PMC10367394; doi:10.1186/s12913-023-09776-y)
Supplement: Supplementary file 2 — Additional file 2: S1 File. Sample size calculation. [file 12913_2023_9776_MOESM2_ESM.docx]

**S1File****. Sample size calculation**

Sample size taken from the Catchment Profile of Western Cluster of EPSA and service delivery point.

| S.N | Branch | Distance from  A. A | Region | Available Zones | Available woredas | Hospitals | Health Center | 30 % of Hsp | 15% of HC |
| --- | --- | --- | --- | --- | --- | --- | --- | --- | --- |
| 1 | Jimma | 358 KM | Oromia | 8 | 81 | 26 | 312 | 8 | 47 |
| 2 | Gambella | 714 KM | Gambella | 3 | 14 | 4 | 27 | 1 | 4 |
| Total sample size of Hospital (30 %) | | | | | | | | 9 |  |
| Total sample size of health center (15 %) | | | | | | | |  | 51 |
| EPSA Branch (70 %) | | | | | | | | 2 |  |
| Total sample size of the study will be | | | | | | | | | 62 |
