# Supplementary material for: Assessment of the magnitude and contributing factors of expired medicines in the public pharmaceutical supply chains of Western Ethiopia
Source: BMC Health Serv Res. 2023 Jul 25;23:791. doi: 10.1186/s12913-023-09776-y (PMC10367394; doi:10.1186/s12913-023-09776-y)
Supplement: Supplementary file 3 — Additional file 3: S2 File. Data Quality Assurance. [file 12913_2023_9776_MOESM3_ESM.docx]

**S2File. Data Quality Assurance**

**Informed consent**

For those who are willing to engage in the study, this information sheet was created. After reading the description of how informed permission was gained, the detailed information that was used in the study was presented.

**Introduction:**  Good day wish you. We are researchers at Jimma University, school of pharmacy, pharmaceutical quality assurance, and regulatory affairs department, and we are doing an assessment of the magnitude and contributing factors of expired medicines in the public pharmaceutical supply chains of western Ethiopia. We are looking for information about the magnitude of expired medicines and their contributing factors.

**Purpose:** The objective of this study was to assess the magnitude of expired medicines and contributing factors, in the public pharmaceutical supply chains in western Ethiopia. After I get your permission, I would like to ask you a series of questions about the expired medicines in your health facility. Such data are fundamental for clinicians, pharmaceutical supply chain agencies, pharmacists, and the patient as a whole to give evidence-based interventions to health planners and care givers to guide future policymakers and would serve as baseline information for further studies in the region and at the national level. Also, I would like to review some documents related to records of expired medicines.

**Risk:** By participating in this data collection, you may sacrifice your time otherwise; you may not face any risk in participating in this data collection.

**Benefits:** By participating in this study, you may not get compensation or benefit right now, but the result of the study will provide information for developing a recommendation on the magnitude of expired medicines and contributing factors.

**Confidentiality:** The performance of individual staff members is not being evaluated. The researcher is not going to take any personal identifiers. The collected data will be analyzed in aggregate without making any personal manipulation. And instead, the researcher will use a coding system to identify your institutions.

**Voluntary participation:** The participation in this study was based on participants voluntary and they have the right to refuse to participate in the study, and the confidentiality of the information gathered from them will be kept and used for this study only. The result of the study will be communicated with your chief pharmaceutical branch managers, or your care givers. If you do not understand something, you may ask questions now and, in the future, that will be being done, contact the investigators on below address.

**Participants Name (Code identifier)**

**Signature**


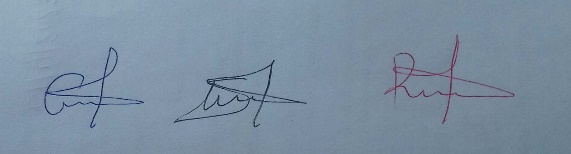


**Researcher name:** Gamachu Diriba, Email: [abbaagammachis0@gmail.com](mailto:abbaagammachis0@gmail.com)

**Supervisors:** Sultan Suleman (PhD, Professor); Email: [sultansulemanl@gmail.com](mailto:sultansulemanl@gmail.com)

Gemmechu Hasen (MSc, Ass. Professor); Email: [gemmechuhasen2009@gmail.com](mailto:gemmechuhasen2009@gmail.com)

Member: Yesuneh Tefera (MSc), Email: [yetefera29@gmail.com](mailto:yetefera29@gmail.com)

***Thank you for your co-operation!***
